# Reference genes expression stability in *Avena sativa* L. during compatible and incompatible interactions with *Puccinia graminis*

**DOI:** 10.1038/s41598-022-22993-5

**Published:** 2022-11-01

**Authors:** Sylwia Sowa, Magdalena Sozoniuk, Joanna Toporowska, Krzysztof Kowalczyk, Edyta Paczos-Grzęda

**Affiliations:** Institute of Plant Genetics, Breeding and Biotechnology, University of Life Sciences in Lublin, Akademicka 13, 20-950 Lublin, Poland

**Keywords:** Agricultural genetics, Reverse transcription polymerase chain reaction, Biotic

## Abstract

A reliable qPCR experiment requires the selection of reference genes with a stable level of expression in a given experimental system. This study attempts to determine the reference genes (RGs) for the *A. sativa–P. graminis* experimental setup. We evaluated nine candidate reference genes in *A. sativa* (oat line Pg4 and the cultivar Kasztan) during compatible and incompatible interactions with different pathotypes of *Puccinia graminis* f. sp. *avenae* in six time points post-inoculation. The identification of genes with high expression stability was performed by four algorithms (geNorm, NormFinder, BestKeeper and ΔCt method). We found that the most appropriate combination of RGs for RT-qPCR data normalization were *HNR* (heterogeneous nuclear ribonucleoprotein 27C) + *EF1A* (elongation factor 1-alpha) + *EIF4A* (eukaryotic initiation factor 4A-3). The worst candidates for normalization in this dataset were *CYP* (cyclophilin) and *TUA* (alpha tubulin). Identified reference genes are suitable candidates for the standardization of gene expression studies in the *A. sativa*–*P. graminis* interaction system and potentially other related pathogens. To date, this is the first report of RGs selection in this pathosystem.

## Introduction

One of the greatest global struggles is the rapid growth of the world’s population, estimated to reach at least 9.8 billion by 2050. Agricultural production must therefore increase as more and more food is needed to provide food security for the rising populations. Cereals classified within the grass family *Poaceae*, also known as *Gramineae,* have a critical role in human nutrition. The world’s population relies on wheat, corn, and rice for its primary caloric intake. However,oat (*Avena sativa* L.) exemplifies the extraordinary nutritional value that cereals can provide^1,2^. Oat grain distinguishes from other cereals by a different nutrient composition. Oat has the lowest polysaccharides content of which starch predominates, it contains large amounts of dietary fibre, in particular, its soluble fraction rich in β-glucans, compounds that are one of the greatest discoveries in recent years^3,4^. Annual oat production reaches 23 million tonnes, with Russia, Canada, Australia and Poland being the largest oat-producing countries^5^.

The global production of cereals is severely affected by rust diseases caused by a specialized group of fungi from the *Pucciniales* order^6^. This diverse and widely distributed group of plant pathogens with more than 7800 species may attack the most frequently sown cereals in the world including oat^7^. Stem rust caused by *Puccinia graminis* Pers*. *f. sp.* avenae* Eriks. & E. Henn*.* regularly occurs world wide and poses a great threat to the oat yield and quality. The use of fungicides is the main way to prevent stem rust development, however it is associated with high costs and is becoming increasingly opposed by consumers. Therefore, genetic resistance is one of the most accepted important modern breeding goals. The life cycle of *P. graminis*, involving sexual and asexual reproduction leads to the emergence of new rust pathotypes and causes high virulence dynamics and phenotypic diversity^8,9^. This efficient fungus adaptability causes rapid loss of effectiveness of current genetic stem rust resistance sources, hence there is urgent need to develop durable plant resistance immune to the pathogen variability.

Successful implementation of durable resistance strategies into plant breeding relies on the understanding of the processes involved in pathogen-host interactions. Accurate quantification and validation of plant genes involved in controlling the disease development is performed by reverse transcription quantitative real-time polymerase chain reaction. Identifying reference genes with stable expression levels will ensure obtaining repeatable and reliable RT-qPCR results. The study attempts to determine the reference genes for the *A.* *sativa–P. graminis* experimental setup. The obtained results will provide valuable data necessary for a comprehensive analysis of gene expression in oat.

## Methods

### Experimental material and rust inoculation

The study material consisted of oat line Pg4 derived from the Hajira cultivar with major gene resistance to stem rust caused by *P. graminis* f. sp. *avenae*^10^ and the polish cultivar Kasztan (Dawid × CHD 1685/84) susceptible to stem rust infection^11^. Seeds of the study genotypes were grown in plug trays filled with a universal substrate containing peat for 10 days in a phytotron at 18 °C for a 16-h photo period.

Two *P. graminis* f. sp. *avenae* pathotypes (D19 2/7, Cz19 3/6, Table 1) were used with a virulence profile defined based on a set of 12 differential oat lines carrying a single stem rust resistance gene (Pg1, Pg2, Pg3, Pg4, Pg6, Pg8, Pg9, Pg10, Pg12, Pg13, Pg15, Pg16)^12^ as well as oat lines ‘Pg-a’, ‘Alpha’ (CI 9221), ‘Omega’ (CI 9139)^13^ and ‘Wisconsin X-1588-2’ (CI 8457). Pathotypes were selected from a collection of single-pustule isolates derived from stem rust populations collected in Poland from 2017 to 2020^9^. Both pathotypes are virulent to ‘Kasztan’. Pg4 line is resistant to Cz19 3/6 and susceptible to D19 2/7. Dried pathotypes were stored in 1.5-ml microfuge tubes at − 70 °C. Before inoculation, urediniospores were heat-shocked for 4 min at 42 °C and multiplied on leaf fragments of the susceptible oat cultivar Kasztan^11^ using the host–pathogen method of Hsam et al.^14^, originally used for *Blumeria graminis* f. sp. *avenae* and modified by Paczos-Grzęda and Sowa^15^. Study was conducted on the first leaves of 10-day-old seedlings. Inoculation was performed in settling towers by spreading urediniospores on plant material at a density of ≈ 200 spores/cm^2^. Pg4 line was inoculated using both *P. graminis* pathotypes and ‘Kasztan’ was inoculated with D19 2/7. Glass slides were placed between the inoculated leaves to monitor the inoculation density. The settling towers were thoroughly cleaned with ethanol between inoculations with different isolates and the room was sprayed with water. Seedlings were incubated in a spore-proof growth chamber at 18 °C with 70% humidity and light intensity of approximately 4 kLx under a 16-h photoperiod.

**Table 1:** Virulence spectrum of *P. graminis* f. sp. *avenae* pathotypes used for inoculation.

Race no	Phenotype code^1^	Virulence to Pg differentials
D19 2/7	TJH	Pg1, Pg2, Pg3, Pg4, Pg8, Pg9, Pg13, Pg16
Cz19 3/6	LSH	Pg1, Pg6, Pg8, Pg9, Pg13, Pg16

^1^Phenotype code based on the standard differentials set^35^.

Plant experiments were performed in accordance with relevant guidelines and regulations.

### RNA extraction and reverse transcription

Plant material for RNA extraction was harvested after 0 (uninoculated ‘Kasztan’ and Pg4), 8, 16, 24, 72 and 96 h post-inoculation (hpi). Total RNA was extracted immediately after sampling in three biological replicates. Each sample comprised of leaves pooled from five different seedlings homogenized in liquid nitrogen using sterile mortar and pestle. The RNA isolation was performed with TRIzol reagent (Invitrogen) according to the manufacturer’s recommendations. The quality and quantity of RNA samples were assessed electrophoretically on 1.5% agarose gel and spectrophotometrically with NanoDrop2000 (Thermo Fisher Scientific Inc., USA). Genomic DNA contamination was removed by DNase I (EURx Ltd., Poland) treatment.

### Two-stepRT-qPCR reactions

The reaction of reverse transcription was performed on 2 µg RNA using oligo d(T)_18_ primer. The cDNA synthesis was performed with NG dART RT kit (EURx Ltd., Poland) in a final volume of 20 µl. The no-reverse transcription control (NRT) was also included in the analysis. Obtained cDNA was diluted and used as a template in the qPCR reactions.

Ten candidate RGs (*ARF, CYP, EF1A, EIF4A, GAPDH, HNR, HSP70, TBP, TUA* and *UBC*) were chosen for expression stability analysis based on the literature review^16,17^. Primers for qPCR reactions were taken from previous studies^18,19^ or designed with PrimerBLAST tool^20^ as indicated in Table S1.

The qPCR reactions were conducted on QuantStudio™ 3 apparatus (Applied Biosystems, USA) using Power Track SYBR Green Master Mix (Thermo Fisher Scientific Inc., USA). The reaction mixture of 20 μL consisted of 20 ng of cDNA, 1 × qPCR Mix and 400 nM of each primer. The reactions were performed according to the following cycling program: 95 °C for 2 min, 40 cycles of 95 °C for 15 s and 60 °C for 1 min. In order to confirm the amplification specificity melting curve analysis was performed after each run with continuous data collection from 60 to 95 °C. Additionally, the existence of single PCR products was further verified by the means of electrophoresis in 2% agarose gel stained with ethidium bromide. All reactions were conducted in three biological replicates with three technical replicates along with no template control (NTC). Standard curves were generated from a fivefold serial dilution of pooled cDNA. Only primer pairs showing amplification efficiency of 90–110%, slope between − 3.6 to − 3.1 and correlation coefficient (R^2^) over 0.99 were used for further analysis.

### Analysis of gene expression stability

The data obtained from RT-qPCR was analysed using a dedicated module from ThermoFisher Cloud (ThermoFisher Scientific). The Cq values were corrected for reaction efficiency. The expression stability of candidate RGs was assessed by geNorm^21^, NormFinder^22^, BestKeeper^23^ and delta Ct method^24^. Overall final ranking was generated according to the method proposed by Velada et al.^25^. Expression analysis of target gene *PAL* (phenylalanine ammonia lyase) was carried out for RGs validation. The RT-qPCR reaction conditions were as described above. The relative expression level of the target gene was calculated using the 2^−∆∆Ct^ method. Statistical significance between untreated and inoculated data subgroups was calculated by Student’s *t* test at P ≤ 0.05.

## Results

The study was conducted on the plant material with a major resistance gene to stem rust (oat line Pg4 with *Pg4* resistance gene) as well as genotype susceptible to rust infection (cultivar Kasztan). The use of stem rust races with defined virulence profiles allowed to stimulate susceptibility (oat line Pg4 and cultivar ‘Kasztan’ infected by D19 2/7 *P. graminis* pathotype) as well as resistance response (oat line Pg4 infected by Cz19 3/6 *P. graminis* pathotype) and observe compatible and incompatible interactions respectively. Studied samples were analysed according to the following experimental sets: full dataset, *A. sativa*–*P. graminis* compatible interaction, *A. sativa*–*P. graminis* incompatible interaction, Pg4 oat line, Kasztan cultivar.

### Primers amplification efficiency and specificity

For reference genes selection, cDNA of tested oat genotypes was used in the qPCR. The primers' specificity was verified by qPCR melting curve analysis and evaluated on 2% agarose gel. Single peaks confirming the specificity of the amplification were generated on the dissociation curves obtained for each primer pair, no signal was detected in the NTC samples. Moreover, single qPCR product bands were observed on agarose gel (Figs. S1, S2).

PCR amplification efficiency evaluated by a standard curve method with a pool of all the cDNAs varied from 96.41% (*CYP*) to 122.4% (*TBP*) and the R^2^ based on linear regression ranged from 0.992 (*HNR, TBP*) to 1 (*ARF*). The primer pair for *TBP* was excluded from the analysis for exceeding efficiency criterion of 110% (Table 2). The Tm values of the primer pairs varied from 83.75 °C (*EF1A*) to 92.13 °C (*CYP*), and the amplicon sizes were between 88 bp (*EIF4A*) and 158 bp (*EF1A*) (Table S1). The raw quantification cycle (Cq) values were estimated for the determination of the gene expression levels. The mean Cq values for analysed samples ranged between 22.79 (*ARF*) and 28.89 (*HNR*) (Fig. S3).

**Table 2:** Parameters derived from RT-qPCR analysis—slope, regression coefficient (R^2^), reaction efficiency and melting temperature of the amplicon (Tm).

Gene	Slope	R^2^	Efficiency (%)	Tm (°C)
*ARF*	− 3.29	1	101.50	85.59
*CYP*	− 3.41	0.996	96.41	92.13
*EF1A*	− 3.19	0.996	105.65	83.75
*EIF4A*	− 3.34	0.996	99.08	86.43
*GAPDH*	− 3.09	0.995	110.21	86.03
*HNR*	− 3.12	0.992	109.1	83.98
*HSP70*	− 3.15	0.999	107.81	86.63
*TBP*	− 2.887	0.992	122.64	83.25
*TUA*	− 3.13	0.994	108.73	84.00
*UBC*	− 3.18	0.999	106.53	84.43
*PAL*	− 3.26	0.993	102.22	86.31

RGs expression stability was assessed with four algorithms, geNorm, NormFinder, BestKeeper and ΔCt method. For each algorithm, RGs were ranked from the most to least stable.

### BestKeeper results

BestKeeper calculates the geometric mean of Cq values and the most stable genes are indicated by high correlation coefficients (r) and low standard deviations (SD)^23^. For all samples analysed in *A. sativa*–*P. graminis* interaction system *HNR*, *EF1A* and *EIF4A* displayed the most stable expression with the highest coefficient of correlation. Moreover, within *EIF4A* results the lowest mean SD (≈ 0.472) was observed. Identical results were obtained for the remaining groups except forthe incompatible interaction dataset, where the best scoring genes were *EIF4A*, *UBC* and *EF1A*. Based on correlation coefficients, *CYP* showed the least stable expression out of all RGs tested in each analysed dataset. The highest mean SD above 1 was observed within *GAPDH* (≈ 1.05) and *TUA* (≈ 1.29) results (Table S2a,b).

### ΔCt results

According to ΔCt analysis based on average standard deviation (mean SD) *HNR* and *EF1A* were ranked as the most stable candidate RGs in the full analysed dataset. Similar results were obtained for compatible interaction samples as well as for Pg4 oat line and Kasztan cultivar samples. For the incompatible interaction subgroup *EF1A* and *EIF4A* were found to be the least variable RGs. The highest mean SD was reported for *TUA* regardless of the dataset (Table S2c).

### geNorm results

GeNorm v3.4 software was used to rank the candidate RGs according to the stability measure (M-value)^21^. The lowest M value always presents the most stably expressed candidate gene with the threshold set at 1.5. All tested genes in analysed datasets showed expression stability below 0.93. When all the samples were combined, the M-value of *ARF*, *EIF4A* and *EF1A* was the lowest, and for *TUA* it was the highest. The results remained very similar when the M-value was measured for the compatible interaction dataset and Pg4. Within incompatible interaction subgroup and samples of cultivar Kasztan, *EF1A* and *HNR* were the most stable among the RGs tested. Out of all genes,*TUA* was found to be the worst performing RG in each analysed sample set (Table S2d).

### NormFinder results

In NormFinder analysis, intra- and intergroup variation within subgroups of a dataset were calculated^22^. In this analysis, SV (stability value) of all tested RGs was below 0.68. The lowest SV characterizing the best candidates for qPCR data normalization was presented by *HNR, EF1A* and *EIF4.* However, they differed in order when groups were evaluated separately. In compatible interaction subgroup as well as Pg4 oat line, *ARF* can also be considered as exhibiting relatively high expression stability. The highest SVs representing high variation in expression was reported for *TUA* and *CYP* (Table S2e).

### Determination of the optimal number of RGs for data normalization

**Figure 1:**
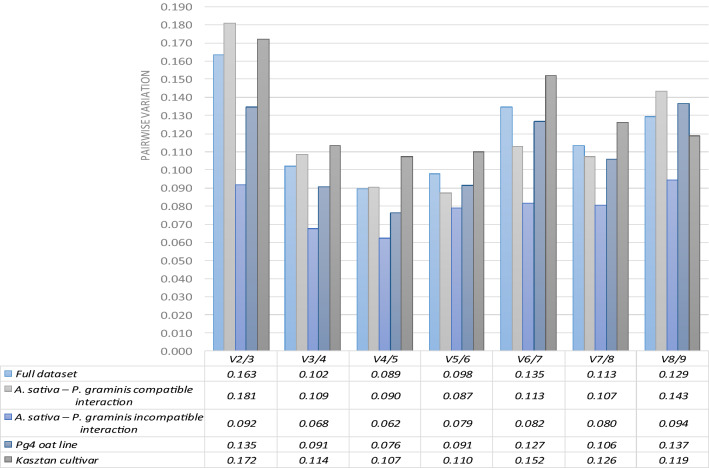
Pairwise variation (V_n_/V_n+1_) analysis performed by geNorm algorithm for determination of the optimal number of RGs for accurate RT-qPCR data normalization. A value < 0.15 indicates no need for additional RG inclusion.

The geNorm algorithm allows the prediction of the optimal number of RGs that should be used for accurate qPCR data normalization by calculating pairwise variation (V_n_/V_n+1_). The V_n_/V_n+1_ value below 0.15 indicates that the inclusion of additional RG does not make any significant contribution to the data analysis. In this study,V_2_/_3_was below 0.15 for incompatible interaction subgroup as well as Pg4 indicating that the two best-performing RGs are enough for sufficient data normalization in these datasets (Fig. 1). If all the samples need to be analysed together, three reference genes are required for optimal normalization, because the V_3_/V_4_ value was lower than 0.15. Similar results were obtained for compatible interaction samples as well as for Kasztan cultivar samples.

According to the comprehensive rankings (Table 3) the most appropriate combination of RGs for full dataset as well as Kasztan cultivar was *HNR* + *EF1A* + *EIF4A*. In the compatible interaction subgroup, *HNR* + *EF1A* + *ARF* were found to be the least variable RGs. In the incompatible interaction subgroup and Pg4 oat line, a pair of best-performing RGs *EF1A* + *EIF4A* was found to be sufficient for obtaining reliable results. *CYP* was shown to be the worst candidate for normalization in the full dataset, Pg4 oat line and Kasztan cultivar. In the compatible and incompatible interaction subgroup, the utmost variation wasdisplayed by *TUA*.

**Table 3:** Comprehensive rankings generated for analysed datasets based on the results obtained with four algorithms. Reference genes recommended for RT-q PCR data normalization are shown in bold.

Number	Full dataset	*A. sativa–P. graminis* compatible interaction	*A. sativa–P. graminis* incompatible interaction	Pg4 oatline	Kasztan cultivar
1	***HNR***	***HNR***	***EF1A***	***HNR***	***HNR***
2	***EF1A***	***EF1A***	***EIF4A***	***EIF4A***	***EF1A***
3	***EIF4A***	***ARF***	*HNR*	*EF1A*	***EIF4A***
4	*ARF*	*EIF4A*	*ARF*	*ARF*	*ARF*
5	*UBC*	*UBC*	*UBC*	*UBC*	*HSP70*
6	*HSP70*	*GAPDH*	*GAPDH*	*GAPDH*	*UBC*
7	*GAPDH*	*HSP70*	*CYP*	*HSP70*	*GAPDH*
8	*TUA*	*CYP*	*HSP70*	*TUA*	*TUA*
9	*CYP*	*TUA*	*TUA*	*CYP*	*CYP*

### Expression analysis of target gene for reference genes validation

To validate the reliability of the selected RGs, the expression analysis of *PAL* gene was performed in experimental samples harvested at various time points post inoculation. For each dataset, both best and worst performing RGs were used to demonstrate how incorrect data normalization may affect obtained results.

When normalization was carried out for Pg4–*P. graminis* compatible and incompatible interaction with most stable RG sets (*HNR* + *EF1A* + *EIF4A* and *HNR* + *EF1A* + *ARF*), no significant difference in expression between untreated (0 hpi) and inoculated data subgroups were observed (Fig. S4). However, when *TUA* and *CYP* were used, a clear over estimation of transcript level was noticed. *PAL* expression level normalized against *CYP* was significantly differentat every time point.

**Figure 2:**
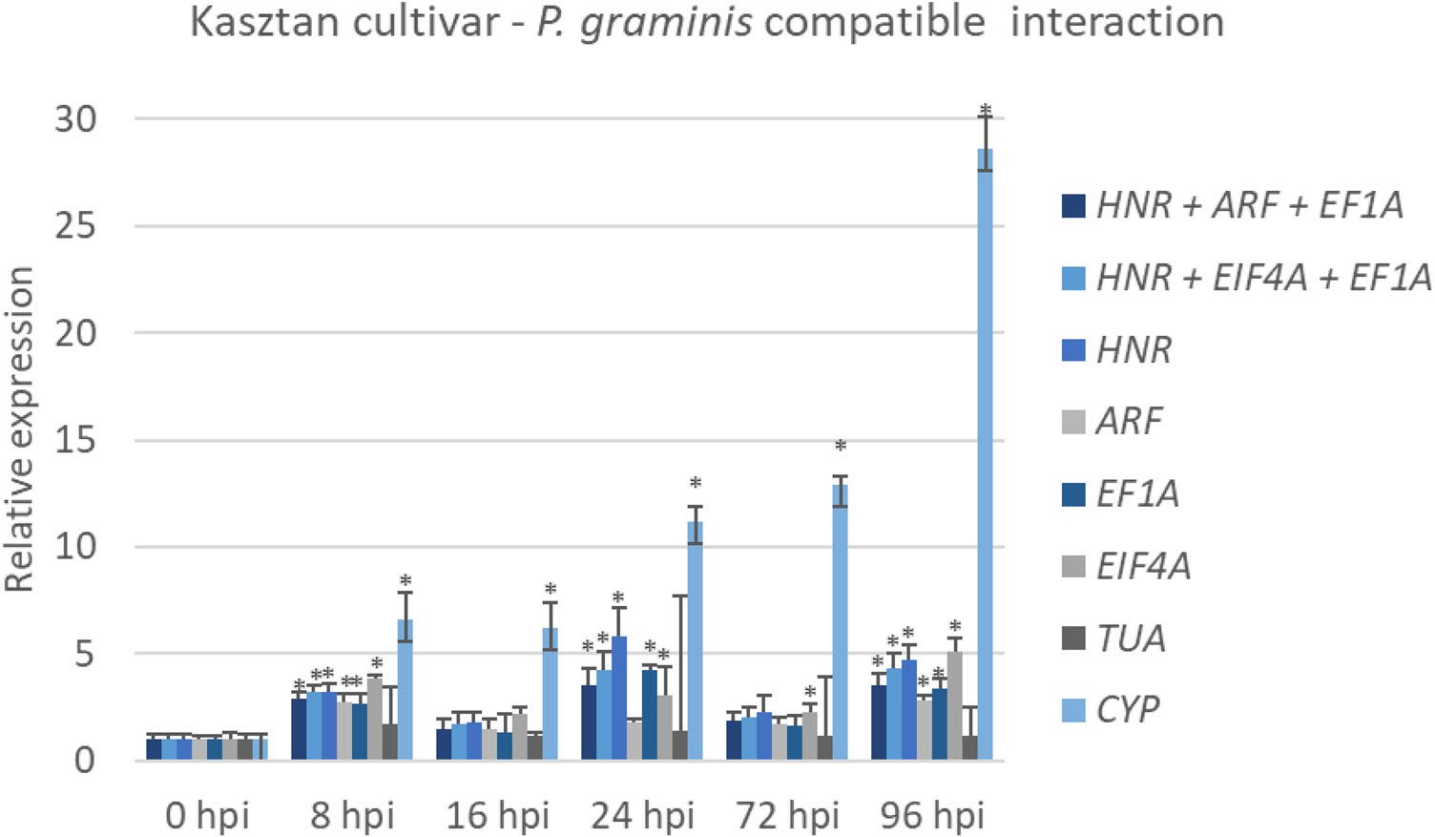
Relative expression of the *PAL* gene in A*. sativa* Kasztan cultivar–*P. graminis* compatible interaction. Analysis was performed against two sets of best performing RGs (*HNR* + *ARF* + *EF1A* and *HNR* + *EIF4A* + *EF1A*) separately or together as well as against worst performing RGs (*CYP* and *TUA*). Data are shown as mean ± SD. * indicate that the difference in expression between untreated (0 h) and inoculated data subgroups is significant at P ≤ 0.05 as determined by Student’s *t* test.

Relative expression of the *PAL* gene in Kasztan cultivar–*P. graminis* incompatible interaction normalized with most stable RG sets was significantly different in 8, 24, and 96 hpi. When *CYP* was used for normalization, a significant difference was observed at every time point. Data normalized against *TUA* showed no significant difference in *PAL* expression level (Fig. 2).

Obtained data confirm substantial inaccuracyin the expression profileswhen RGs selection is not carried out properly.

## Discussion

Identification of reference genes exhibiting stable expression has been an objective of several recent studies carried out in *Avena sativa*^26–28^ and *Avena fatua*^18^. Neither of them, however, investigated the effect of fungal infection on candidate RGs expression. Therefore, the goal of the present study was to evaluate the set of ten potential RGs in terms of their expression stability in the plant-pathogen system consisting of *A. sativa* and *P. graminis*.

In our study, we tested the candidate RGs stability during *A. sativa*–*P. graminis* compatible and incompatible interactions throughout six time points post-inoculation. While analyzing the full dataset we found that the most appropriate combination of RGs for RT-qPCR data normalization was *HNR* + *EF1A* + *EIF4A*. In the compatible interaction subgroup data normalization also required the use of three internal controls (*HNR* + *EF1A* + *ARF*), whereas in the incompatible interaction subgroup, just a pair of best-performing RGs (*EF1A* + *EIF4A*) was found to be sufficient for obtaining reliable results. Likewise, high expression stability of *HNR* and *EIF4A* across all tested *A. sativa* samples was reported by Yang et al.^18^. It is also worth mentioning, that *HNR* and *ARF* used for normalization separately in the gene expression experiment, showed comparable output to the results obtained by three RGs normalisation. This might be an alternative to consider for large experimental studies or under budget constraints.

Duan et al.^27^ emphasized the effect of specific sample subsets on the proper RGs selection. In their study, the pair of best-performing RGs in salt-stressed oat samples included *EF1A* and *TBP*. However, in the sample set comprising of different tissues, *TBP* displayed medium expression stability. The highest-ranked RGs chosen in this subgroup were *EF1A* and *PP2A*. Similar conclusions were reported by Tajti et al.^28^ who conducted expression analyses in leaves and roots of oat seedlings subjected to various abiotic stresses. The comprehensive rankings generated for specific datasets showed high variance in RGs order. In some cases, the same RG was ranked either at first or last position, depending on the experimental dataset (e.g., *GAPDH* in salt-stressed leaves and in cold stressed leaves, respectively).

In our study, sample subgroups consisted of more homogeneous material, therefore there is little variance in the order of RGs among different sample sets. In fact, in four out of five datasets the same three RGs were reported to have the most stable expression, that is *HNR*, *EF1A* and *EIF4A*. The above mentioned study by Duan et al.^27^ revealed poor expression stability of commonly used RGs (*GAPDH*, *ACT* and *TUA*) in oat specimens. This is in concordance with our results which demonstrated medium-to-low expression stability of *GAPDH* and *TUA* in the tested material. Likewise, *GAPDH* was reported as the least stable RG in developing oat seeds and endosperms in the study of Yang et al.^18^. Nonetheless, Wise et al.^29^ in their study on methods of enhancing crown rust resistance in cultivated oat by application of plant defence activators selected *GAPDH* in combination with *HSP70* as stable internal controls for RT-qPCR data normalization. Moreover, *GAPDH* was chosen as a stable endogenous control in the research on oat–crown rust interaction carried out by Montilla-Bascón et al.^30^. Worth mentioning, however, is the fact that the aforementioned study evaluated the stability of only four candidate RGs. If the larger panel of potential RGs had been tested, it is likely that different gene would have been selected as the internal control.

Experiments aiming at elucidation of the molecular basis of resistance should investigate both compatible and incompatible interactions between host and pathogen^31^. Different interaction types may induce various alternations to the host transcriptome, therefore validation of RGs stability in both of them is crucial for proper data analysis. There have been several reports assessing RGs stability in plant-pathogen systems involving compatible- and incompatible interactions. Albuquerque et al.^32^ studied RGs expression stability in tomato during interaction with *Ralstonia solanacearum* isolates displaying contrasting virulence profiles. Interestingly, identified most stable RGs differed between compatible and incompatible interaction analysis groups (*PDS* + *ACT* vs *TIP41* + *EF1A*, respectively). Research conducted by Monteiro et al.^33^ in cultivated grapevine–*Plasmopara viticola* pathosystem also revealed the need of utilizing different sets of RGs for studying compatible and incompatible interactions. Here, we obtained more congruous results among compatible and incompatible interaction datasets, with only one out of the three best RGs being different.

The literature shows that resistance-associated genes expression differs during compatible and incompatible interactions^34^ Those differently expressed genes might be employed in fungal disease resistance screening. However, in order to avoid erroneous data interpretation utilization of carefully selected RGs is required. Here, we propose the use of identified sets of RGs to be explored in the further investigations of *A. sativa- P. graminis* and potentially other cereal pathosystems.

## Supplementary Information

Supplementary Information.

## Data Availability

The datasets generated during and/or analysed during the current study are available from the corresponding author upon reasonable request.

## Abbreviations

RG: Reference gene
RT-qPCR: Reverse transcription quantitative real-time polymerase chain reaction
*ARF*: ADP-ribosylation factor
*CYP*: Cyclophilin
*EF1A*: Elongation factor 1-alpha
*EIF4A*: Eukaryotic initiation factor 4A-3
*GAPDH*: Glyceraldehyde-3-phosphate dehydrogenase
*HNR*: Heterogeneous nuclear ribonucleoprotein 27C
*HSP70*: Heat shock protein 70
*TUA*: Alpha tubulin
*UBC*: Ubiquitin conjugating enzyme (E2)
*PAL*: Phenylalanine ammonia lyase
