# Supplementary material for: Reference genes expression stability in Avena sativa L. during compatible and incompatible interactions with Puccinia graminis
Source: Sci Rep. 2022 Nov 1;12:18369. doi: 10.1038/s41598-022-22993-5 (PMC9626582; doi:10.1038/s41598-022-22993-5)

**Tab. S1** Primer sequences for candidate reference genes.

| Gene symbol | Gene description | Primers/sequence source | Primer sequence 5’→3’ | Amplicon size (bp) |
| --- | --- | --- | --- | --- |
| *ARF* | ADP-ribosylation factor | XM_044532078.1 PREDICTED: *Triticum aestivum* ADP-ribosylation factor 1-like (LOC123111312), transcript variant X2, mRNA | F: GCCATGAATGCGGCTGAAA | 113 |
|  |  |  | R: AGTCCCTCGTACAATCCCTC |  |
| *CYP* | Cyclophilin | EU035525.*1 Triticum aestivum* cyclophilin mRNA, complete cds | F: ATGGCCAACCCCAAGGTCT | 145 |
|  |  |  | R: TCTTGCCCACGCCCTTCTC |  |
| *EF1A* | Elongation factor 1-alpha | (Wrzesińska et al. 2016)  KT153026.1 *Avena fatua* elongation factor 1-alpha (EF1) mRNA, partial cds | F: CAGGCAGATGATCTGCTGCT | 158 |
|  |  |  | R: CCTCAAAGCCAGAGATTGGAA |  |
| *EIF4A* | Eukaryotic initiation factor 4A-3, | (Yang et al. 2020)  Locus_3892_Transcript_3/4_Confi- dence_0.667_Length_1160 | F: TCTCGCAGGATACGGATGTCG | 88 |
|  |  |  | R: TCCATCGCATTGGTCGCTCT |  |
| *GAPDH* | Glyceraldehyde-3-phosphate dehydrogenase | (Wrzesińska et al. 2016)  KT153027.1 *Avena fatua* glyceraldehyde-3-phosphate dehydrogenase (GAPDH) mRNA, partial cds | F: CGTCAGGAACCCTGAAGAAA | 146 |
|  |  |  | R: CTTTGCTAGGGGCTGAAATG |  |
| *HNR* | Heterogeneous nuclear ribonucleoprotein 27C | (Yang et al. 2020)  Locus_4951_Transcript_1/4_Confi- dence_0.667_Length_1455 | F: ATTGGGTTTGTCACTTTCCGTAG | 134 |
|  |  |  | R: CTTGGAGGGTGTCTCGCATCT |  |
| *HSP70* | Heat shock protein | XM_003578850.3 PREDICTED: *Brachypodium distachyon* heat shock cognate 70 kDa protein 2 (LOC100843308), mRNA | F: ATCGACGCTGCCATTCAGTG | 109 |
|  |  |  | R: TCTTGGCGATGATGGGGTTG |  |
| *TBP* | TATA box binding protein | L07604.1 *Triticum aestivum* DNA-binding protein (TFIID) mRNA, complete cds | F: GATGAGGCAGCCGAAGATTG | 102 |
|  |  |  | R: TCCAAAGTCAACCATCATTGCT |  |
| *TUA* | Tubulin alpha | KT153029.1 *Avena fatua* alpha-tubulin (TUA) mRNA, partial cds | F: CGCCGCTCCCTTGATATTGA | 151 |
|  |  |  | R: TTGGGTAGGGCACCAGATTG |  |
| *UBC* | Ubiquitin conjugating enzyme (E2) | XM_044536383.1 PREDICTED*: Triticum aestivum* ubiquitin-conjugating enzyme E2 2 (LOC123115130), mRNA | F: CTGTGCGACCCGAATCCAAA | 103 |
|  |  |  | R: GCTCCACGATCTCACGAACT |  |
| *PAL* | Phenylalanine ammonia lyase | (Tajti et al. 2021)  MT150275.1  *Triticum aestivum* phenylalanine ammonia-lyase (PAL) mRNA, complete cds | F: GCAACTTCCAGGGCACCC | 95 |
|  |  |  | R: CTCCGAGAACTGAGCGAACAT |  |


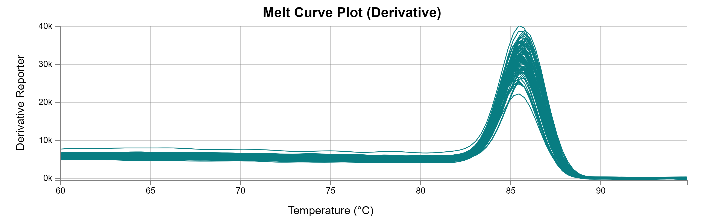

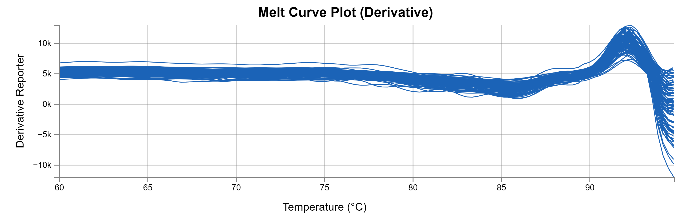


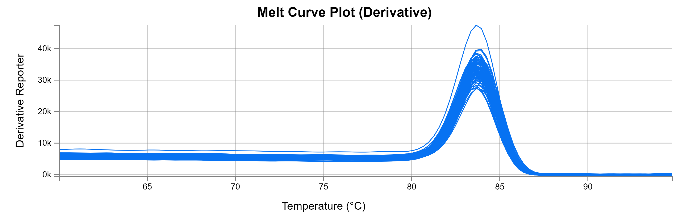

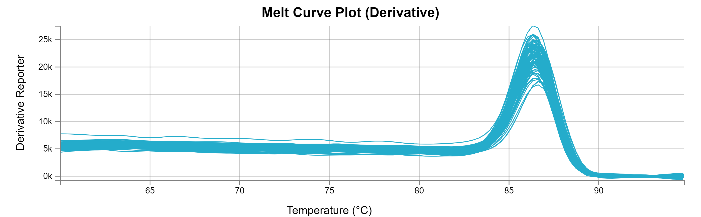


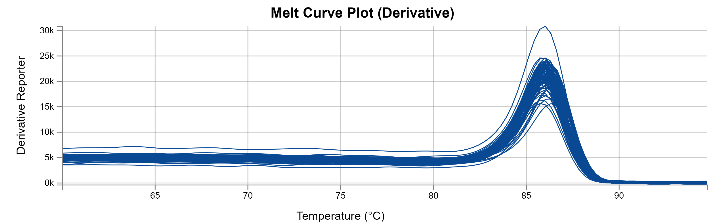

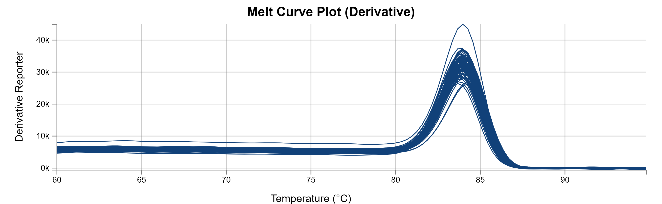

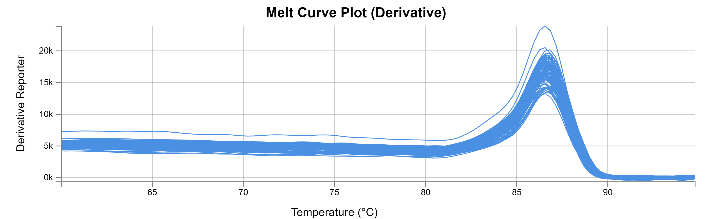

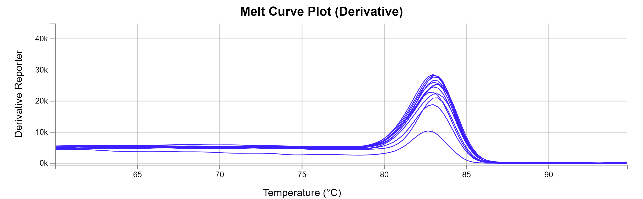


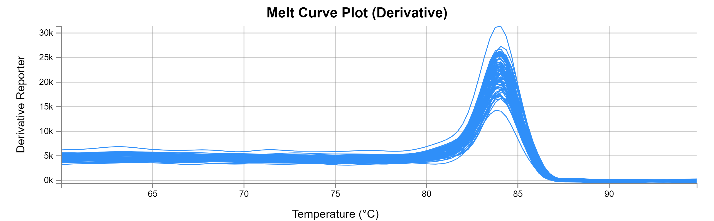

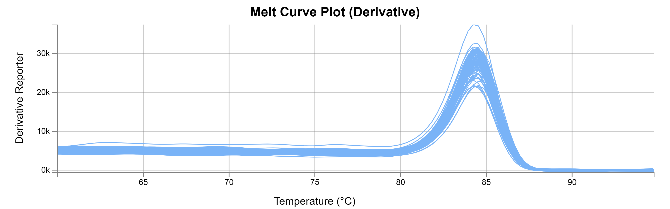


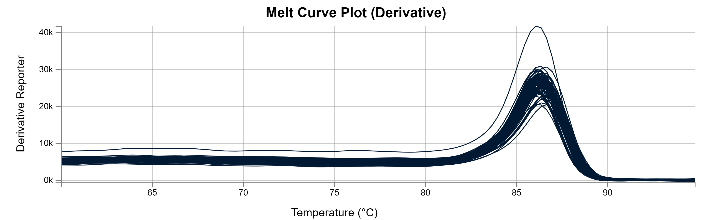


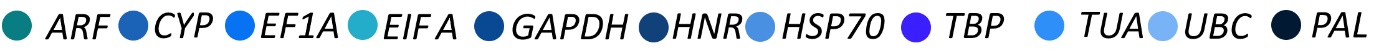


**Fig. S1** Melt curves forRT‐qPCR amplification of tested reference genes.

*ARF* - ADP-ribosylation factor, *CYP* - cyclophilin, *EF1A* - elongation factor 1-alpha, *EIF4A* - eukaryotic initiation factor 4A-3, *GAPDH* - glyceraldehyde-3-phosphate dehydrogenase, *HNR* - heterogeneous nuclear ribonucleoprotein 27C, *HSP70* - heat shock protein, *TUA* - alpha tubulin, *UBC* - ubiquitin conjugating enzyme (E2),*PAL* - Phenylalanine ammonia lyase.


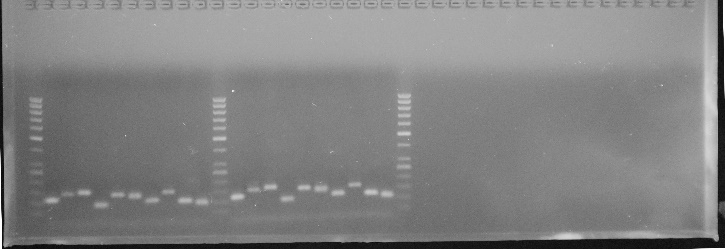

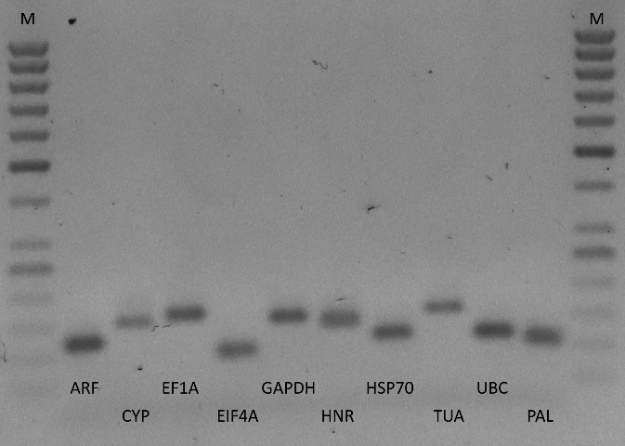


**Fig. S2** qPCR products of candidate reference genes and the gene of interest (*PAL* - Phenylalanine ammonia lyase) on agarose gel.

*ARF*-ADP-ribosylation factor, *CYP* -cyclophilin, *EF1A* - elongation factor 1-alpha,*EIF4A* -eukaryotic initiation factor 4A-3, *GAPDH* - glyceraldehyde-3-phosphate dehydrogenase,*HNR* - heterogeneous nuclear ribonucleoprotein 27C,*HSP70* - heat shock protein,*TUA* - alpha tubulin,*UBC* - ubiquitin conjugating enzyme (E2).

M- 50bp Gene Ruler DNA ladder (Thermo Fisher Scientific Inc., USA).


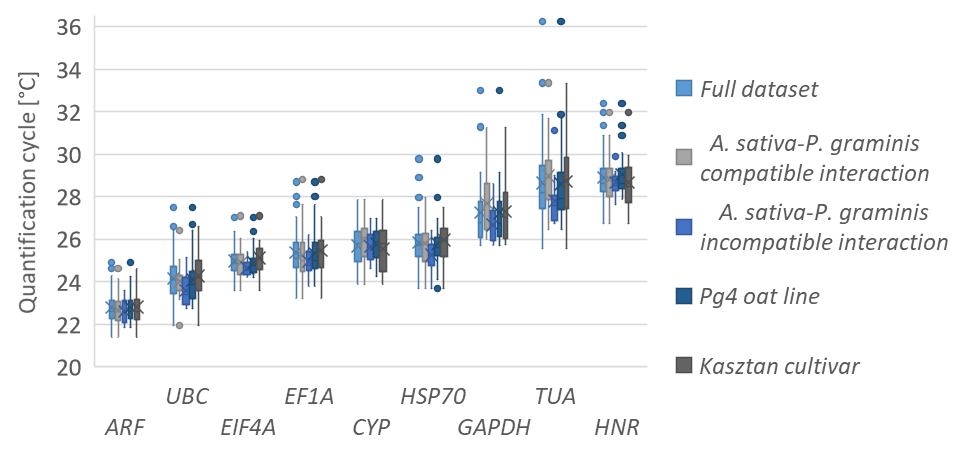


**Fig. S3**Cq values for nine candidate reference genes across experimental samples. A line across the box is depicted as the median. The box indicates the 25th and 75th percentiles, the dots represent outlier values.

*ARF* - ADP-ribosylation factor, *CYP* - cyclophilin, *EF1A* - elongation factor 1-alpha, *EIF4A* - eukaryotic initiation factor 4A-3, *GAPDH* - glyceraldehyde-3-phosphate dehydrogenase, *HNR* - heterogeneous nuclear ribonucleoprotein 27C, *HSP70* - heat shock protein, *TUA* - alpha tubulin, *UBC* - ubiquitin conjugating enzyme (E2).

**Tab. S2** Expression stability of tested RGs determined by BestKeeper,ΔCt, geNorm and NormFinder algorithms for full dataset, compatible interaction dataset, incompatible interaction dataset, Pg4 oat line dataset and Kasztan cultivar dataset.

|  |  | <:::: Least stable genes Most stable genes ::::> | | | | | | | | |
| --- | --- | --- | --- | --- | --- | --- | --- | --- | --- | --- |
| **a.** |  | ***CYP*** | ***HSP70*** | ***ARF*** | ***TUA*** | ***UBC*** | ***GAPDH*** | ***EIF4A*** | ***EF1A*** | ***HNR*** |
| **BestKeeper**  **Correlation coefficients (r)** | Full dataset | 0.665 | 0.889 | 0.887 | 0.928 | 0.894 | 0.911 | 0.947 | 0.956 | 0.963 |
|  | *A. sativa* – *P. graminis* compatible interaction | 0.815 | 0.916 | 0.943 | 0.937 | 0.916 | 0.95 | 0.957 | 0.973 | 0.976 |
|  | *A. sativa* – *P. graminis* incompatible interaction | 0.63 | 0.656 | 0.844 | 0.757 | 0.927 | 0.875 | 0.928 | 0.925 | 0.903 |
|  | Pg4 oatline | 0.686 | 0.898 | 0.914 | 0.931 | 0.95 | 0.932 | 0.963 | 0.947 | 0.978 |
|  | Kasztan cultivar | 0.658 | 0.877 | 0.848 | 0.925 | 0.796 | 0.873 | 0.95 | 0.974 | 0.987 |
|  | Mean | 0.6908 | 0.8472 | 0.8872 | 0.8956 | 0.8966 | 0.9082 | 0.949 | 0.955 | 0.9614 |
|  |  |  |  |  |  |  |  |  |  |  |
| **b.** |  | ***TUA*** | ***GAPDH*** | ***EF1A*** | ***HSP70*** | ***HNR*** | ***UBC*** | ***CYP*** | ***ARF*** | ***EIF4A*** |
| **BestKeeper**  **standard deviation (SD)** | Full dataset | 1.379 | 1.122 | 0.849 | 0.820 | 0.789 | 0.819 | 0.794 | 0.582 | 0.505 |
|  | *A. sativa* – *P. graminis* compatible interaction | 1.581 | 1.245 | 0.951 | 0.935 | 0.931 | 0.745 | 0.771 | 0.591 | 0.552 |
|  | *A. sativa* – *P. graminis* incompatible interaction | 0.798 | 0.629 | 0.469 | 0.553 | 0.492 | 0.634 | 0.568 | 0.439 | 0.300 |
|  | Pg4 oatline | 1.399 | 1.093 | 0.791 | 0.778 | 0.736 | 0.795 | 0.698 | 0.550 | 0.451 |
|  | Kasztan cultivar | 1.316 | 1.174 | 0.934 | 0.871 | 0.897 | 0.838 | 0.971 | 0.641 | 0.554 |
|  | Mean | 1.295 | 1.053 | 0.799 | 0.791 | 0.769 | 0.766 | 0.760 | 0.561 | 0.472 |
|  |  |  |  |  |  |  |  |  |  |  |
| **c.** |  | ***TUA*** | ***CYP*** | ***GAPDH*** | ***HSP70*** | ***UBC*** | ***ARF*** | ***EIF4A*** | ***EF1A*** | ***HNR*** |
| Δ**Ct**  **average standard deviation (mean SD)** | Full dataset | 1.26 | 1.07 | 0.97 | 0.84 | 0.79 | 0.77 | 0.74 | 0.7 | 0.69 |
|  | *A. sativa* – *P. graminis* compatible interaction | 1.38 | 0.94 | 0.94 | 0.82 | 0.77 | 0.76 | 0.76 | 0.69 | 0.67 |
|  | *A. sativa* – *P. graminis* incompatible interaction | 0.96 | 0.73 | 0.64 | 0.75 | 0.55 | 0.53 | 0.52 | 0.5 | 0.53 |
|  | Pg4 oatline | 1.3 | 1.01 | 0.92 | 0.8 | 0.68 | 0.72 | 0.71 | 0.66 | 0.61 |
|  | Kasztan cultivar | 1.2 | 1.16 | 1.05 | 0.9 | 0.94 | 0.84 | 0.76 | 0.73 | 0.7 |
|  | Mean | 1.22 | 0.982 | 0.904 | 0.822 | 0.746 | 0.724 | 0.698 | 0.656 | 0.64 |
|  |  |  |  |  |  |  |  |  |  |  |
| **d.** |  | ***TUA*** | ***CYP*** | ***GAPDH*** | ***HSP70*** | ***UBC*** | ***HNR*** | ***EF1A*** | ***ARF*** | ***EIF4A*** |
| **geNorm**  **stability value (M)** | Full dataset | 0.870 | 0.757 | 0.673 | 0.542 | 0.482 | 0.452 | 0.429 | 0.271 | 0.271 |
|  | *A. sativa* – *P. graminis* compatible interaction | 0.852 | 0.627 | 0.708 | 0.538 | 0.502 | 0.476 | 0.452 | 0.244 | 0.244 |
|  | *A. sativa* – *P. graminis* incompatible interaction | 0.597 | 0.448 | 0.380 | 0.508 | 0.316 | 0.217 | 0.217 | 0.267 | 0.289 |
|  | Pg4 oatline | 0.824 | 0.688 | 0.605 | 0.472 | 0.407 | 0.358 | 0.383 | 0.232 | 0.232 |
|  | Kasztan cultivar | 0.920 | 0.839 | 0.746 | 0.596 | 0.524 | 0.237 | 0.237 | 0.467 | 0.432 |
|  | Mean | 0.812 | 0.672 | 0.622 | 0.531 | 0.446 | 0.348 | 0.343 | 0.296 | 0.293 |
|  |  |  |  |  |  |  |  |  |  |  |
| **e.** |  | ***TUA*** | ***CYP*** | ***GAPDH*** | ***HSP70*** | ***UBC*** | ***ARF*** | ***EIF4A*** | ***EF1A*** | ***HNR*** |
| **NormFinder**  **stability value**  **(SV)** | Full dataset | 0.335 | 0.298 | 0.259 | 0.221 | 0.186 | 0.199 | 0.153 | 0.127 | 0.147 |
|  | *A. sativa* – *P. graminis* compatible interaction | 0.403 | 0.301 | 0.269 | 0.247 | 0.223 | 0.201 | 0.210 | 0.121 | 0.102 |
|  | *A. sativa* – *P. graminis* incompatible interaction | 0.508 | 0.363 | 0.245 | 0.460 | 0.255 | 0.219 | 0.217 | 0.215 | 0.085 |
|  | Pg4 oatline | 0.556 | 0.567 | 0.397 | 0.318 | 0.278 | 0.255 | 0.215 | 0.268 | 0.119 |
|  | Kasztan cultivar | 0.559 | 0.676 | 0.532 | 0.451 | 0.527 | 0.371 | 0.305 | 0.248 | 0.180 |
|  | Mean | 0.472 | 0.441 | 0.340 | 0.339 | 0.294 | 0.249 | 0.220 | 0.196 | 0.127 |

**Fig. S4** Relative expression of the *PAL* gene following *P. graminis* f. sp. *avenae* inoculation. Analysis was performed for *A. sativa* Pg4 - *P. graminis* compatible (a) and incompatible (b) interaction against two sets of best performing RGs (*HNR* + *ARF* + *EF1A* and *HNR* + *EIF4A* + *EF1A*) separately or together as well as against worst performing RGs (*CYP* and *TUA*).

Data are shown as mean ± SD.

* indicate that the difference in expression between untreated (0 h) and inoculated data subgroups is significant at P ≤ 0.05 as determined by Student’s t-test.


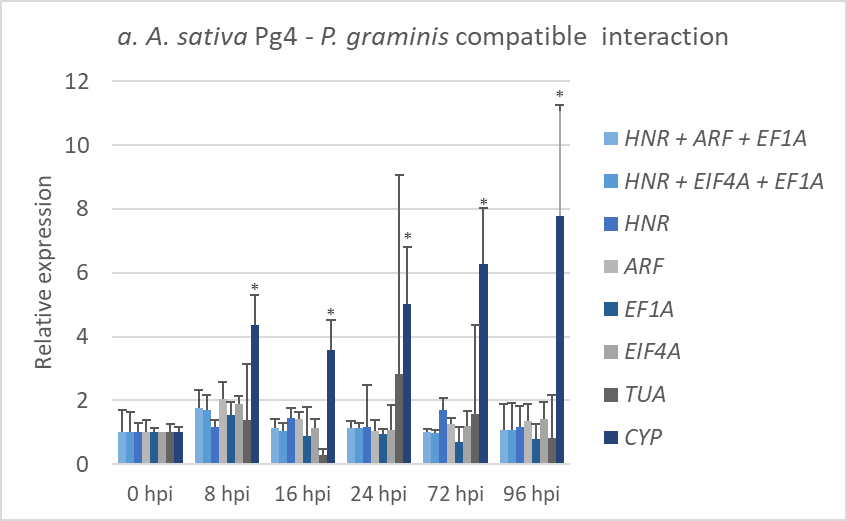


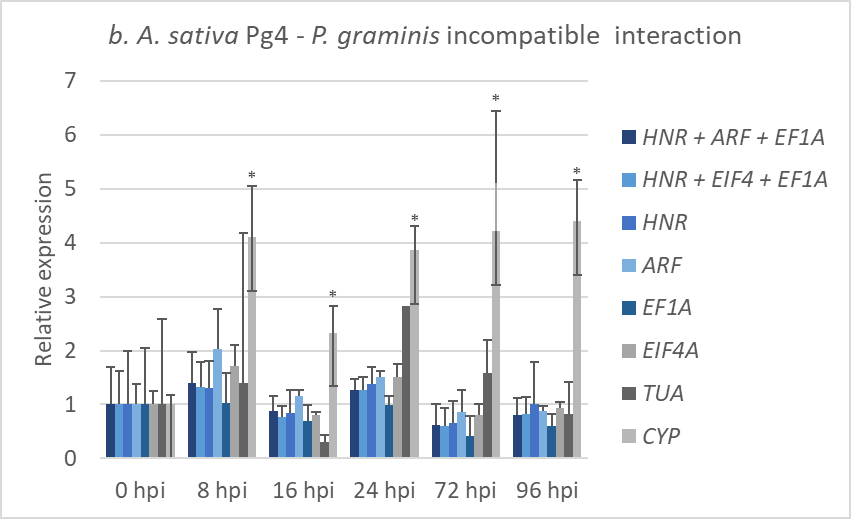

Supplement: Supplementary file 1 — Supplementary Information. [file 41598_2022_22993_MOESM1_ESM.docx]
